# Determinants of Long-Term Outcome in Patients Undergoing Simultaneous Resection of Synchronous Colorectal Liver Metastases

**DOI:** 10.1371/journal.pone.0105747

**Published:** 2014-08-27

**Authors:** Qi Lin, Qinghai Ye, Dexiang Zhu, Ye Wei, Li Ren, Lechi Ye, Qingyang Feng, Pingping Xu, Peng Zheng, Minzhi Lv, Jia Fan, Jianmin Xu

**Affiliations:** 1 Department of General Surgery, Zhongshan Hospital, Fudan University, Shanghai, China; 2 Department of Liver Surgery, Zhongshan Hospital, Fudan University, Shanghai, China; 3 Department of Biostatistics, School of Public Health, Fudan University, Shanghai, China; The First Affiliated Hospital of Nanjing Medical University, China

## Abstract

**Background:**

It remains unclear which patients can benefit from simultaneous resection of synchronous colorectal liver metastases (SCRLMs). This study aimed to examine the prognostic value of patient- and tumor-related factors in predicting long-term outcomes of patients undergoing simultaneous resection of SCRLMs and to help patients select a suitable therapeutic regimen and proper surveillance.

**Methods:**

Clinicopathological and outcome data of 154 consecutive SCRLM patients who underwent simultaneous resection between July 2003 and July 2013 were collected from our prospectively established SCRLM data and analyzed with univariate and multivariate methods, and the prognostic index (PI) was formulated based on the regression coefficients (β) of the Cox model. The patients were classified into high- and low-risk groups according to the PI value; the cut-off point was the third quartile.

**Results:**

The 5-year overall survival rate was 46%, and the 5-year disease-free survival rate was 35%. Five factors were found to be independent predictors of poor overall survival (OS) by multivariate analysis: positive lymph node status, vascular invasion, *BRAF* mutation, the distribution of bilobar liver metastases (LMs) and non-R0 resection of LMs. Compared to low PI (≤5.978), high PI (>5.978) was highly predictive of shorter OS. Three factors were found to be independent predictors of poor disease-free survival (DFS) by multivariate analysis: tumor deposits, *BRAF* mutation and bilobar LM distribution. We also determined the PI for DFS. Compared to low PI (≤2.945), high PI (>2.945) was highly predictive of shorter DFS.

**Conclusions:**

Simultaneous resection of SCRLM may lead to various long-term outcomes. Patients with low PI have longer OS and DFS, while those with high PI have shorter OS and DFS. Thus, patients with high PI may receive more aggressive treatment and intensive surveillance, This model needs further validation.

## Introduction

Colorectal cancer (CRC) is the third most commonly diagnosed cancer in males and the second in females, with over 1.2 million new cancer cases and 608,700 deaths estimated to have occurred worldwide in 2008 [1]. Approximately 25% of CRC patients present with synchronous colorectal liver metastases (SCRLMs) at the time of initial diagnosis, and 25∼50% of patients subsequently develop metachronous colorectal liver metastases (MCRLMs) at the time of disease recurrence [2]. The median survival is 2.3 to 21.3 months for patients with unresectable liver metastases (LMs) [3]. A subset of patients with either SCRLM or MCRLM benefit from radical surgery and may even achieve a cure; approximately 25∼50% of patients with surgically resected colorectal liver metastasis (CRLM) survive 5 or more years [4]–[7].

Unfortunately, the optimal surgical strategy for patients presenting with SCRLM remains controversial. Traditionally, patients have undergone a two-stage procedure with resection of the primary colorectal tumor followed by chemotherapy and subsequent liver resection. Presently, an increasing number of studies have demonstrated that simultaneous resection is an acceptable and safe option due to advances in the surgical technique of liver resection and enhancements in anesthesia and critical care [3], [4], [8], [9]. This strategy has potential benefits in terms of cost and quality of life, particularly as using contemporary chemotherapy regimens may damage the liver parenchyma and leave patients ineligible for surgical resection [3].

Several previous studies have reported prognostic factors for patients undergoing two-stage resection [5], [8]–[10]; however, few have examined the factors that influence long-term outcome in patients exclusively undergoing simultaneous resection, and the sample size was very small [4], [11].

This study aimed to examine the prognostic value of patient- and tumor-related factors in predicting long-term outcomes in a cohort of prospectively collected patients undergoing simultaneous resection of SCRLMs and to help patients to select a suitable therapeutic regimen and proper surveillance.

## Patients and Methods

### Inclusion Criteria

We reviewed our prospectively collected SCRLM database between July 2003 and July 2013 and identified 154 consecutive patients treated with simultaneous curative surgery for initially resectable SCRLM. The selection criteria for simultaneous surgery have been reported previously: [12] expected radical resection of primary disease and margin-negative resection of liver LMs (R0), no unresectable extrahepatic disease and adequate predicted volume of the post-resection hepatic remnant. All deaths occurred were due to colorectal cancer. Patients who underwent previous hepatic resections or ablations of the CRLM, experienced perioperative death or had incomplete materials were excluded from this study. The study was approved by the institutional review board of Zhongshan Hospital. All of the patients provided written consent.

### Data Collection

Clinicopathological data for each patient were collected from our prospectively collected SCRLM database. The tumors were staged using the 7th edition of The International Union Against Cancer (UICC)/American Joint Committee on Cancer (AJCC) Tumor, Node, Metastasis (TNM) classification system [13]. *KRAS* and *BRAF* mutations were detected by pyrosequencing, and the timing of perioperative chemotherapy was recorded. The follow-up regimen included routine computed tomography of the chest, abdomen and pelvis and regular colonoscopic surveillance. Disease recurrence was recorded on the basis of clinical, endoscopic or radiological findings at the time of diagnosis. The date of last follow-up, vital status and recurrence of LM were determined and recorded for all patients. Overall survival (OS) was calculated from the date of diagnosis to the date of death due to CRC or last follow-up. Disease-free survival (DFS) was measured from the date of surgery until the date of documented disease recurrence.

### Statistical Analysis

Summary statistics were obtained using established methods and are presented as percentages or median values with standard deviations. Categorical data are summarized as percentages and were analyzed using chi-squared analysis or Fisher's exact test. OS and DFS were analyzed using the Kaplan-Meier method; survival curves were compared using the log-rank test. Univariate and multivariate analyses were performed using the Cox proportional hazards model; significant prognostic factors in univariate analysis were entered into the Cox proportional hazards model using stepwise selection to identify independent predictors. The prognostic index (PI) of patients was calculated based on the regression coefficients (β) of the Cox model: PI = β1×1+β2×2 +…+ βn×n. The patients were classified into high- and low-risk groups according to the PI value; the cut-off point was the third quartile [14]–[16]. All of the statistical analyses were performed using SPSS 16.0 software (SPSS, Chicago, IL, USA). Two-sided P-values were calculated, and P<0.05 was considered significant.

## Results

### Clinicopathological Characteristics of the Patients

From July 2003 to July 2013, a total of 189 patients underwent simultaneous CRC and hepatic resection; 35 patients were excluded because of benign hepatic disease, incomplete material or the presence of other malignant tumors. Detailed clinicopathological data of the 154 patients who underwent simultaneous resection of SCRLMs are shown in Table 1. The majority of patients were male (57.8%) and younger than 60.0 years (66.2%). Most patients presented with a primary colon tumor (69.5%). The average number of metastases was 1.97±1.30 (1.0–8.0). The average size of the largest metastasis was 3.86±2.45 cm (0.5 cm–15 cm).

**Table 1:** Clinicopathological characteristics of the 154 patients who underwent simultaneous resection of primary and synchronous colorectal liver metastases.

Variable		N (%)
Clinical characteristics		
Age	≤60	102 (66.2)
	>60	52 (33.8)
Gender	Male	89 (57.8)
	Female	65 (42.2)
Primary tumor site	Colon	107 (69.5)
	Rectum	47 (30.5)
Histological type	Adenocarcinoma	130 (84.4)
	Mucinous adenocarcinoma	24 (15.6)
Tumor differentiation	Well, moderate	82 (53.2)
	Poor and others	72 (46.8)
Primary tumor (T) stage	T1	3 (1.9)
	T2	5 (3.2)
	T3	25 (16.2)
	T4	121 (78.7)
Primary nodal (N) stage	N0	58 (37.7)
	N1	68 (44.1)
	N2	28 (18.2)
Tumor deposits	Negative	90 (58.4)
	Positive	64 (41.6)
Vascular invasion	Negative	127 (82.5)
	Positive	27 (17.5)
Nerve invasion	Negative	135 (87.7)
	Positive	19 (12.3)
No. of metastases	≤3	129 (83.8)
	≥4	25 (16.2)
Largest metastasis	<5 cm	111 (72.1)
	≥5 cm	43 (27.9)
Tumor distribution	Unilobar	109 (70.8)
	Bilobar	45 (29.2)
Extrahepatic metastases	No	147 (95.5)
	Yes	7 (4.5)
Resection margin	R0	146 (94.8)
	R1	8 (5.2)
CEA	≤5 ng/ml	87 (56.5)
	>5 ng/ml	67 (43.5)
*KRAS* status	Wild type	111 (72.1)
	Mutated	43 (27.9)
*BRAF* status	Wild type	140 (90.9)
	Mutated	14 (9.1)
Primary tumor operation	Right hemicolectomy	58 (37.7)
	Left hemicolectomy	51 (33.1)
	Rectectomy	45 (29.2)
Liver operation	Wedge resection	115 (74.7)
	Hemihepatectomy	28 (18.2)
	Extended hepatectomy	3 (1.9)
	Unknown extent of hepatic resection	8 (5.2)
Treatment	Perioperative chemotherapy	38 (24.7)
	Postoperative chemotherapy	154 (100)

**Figure 1:**
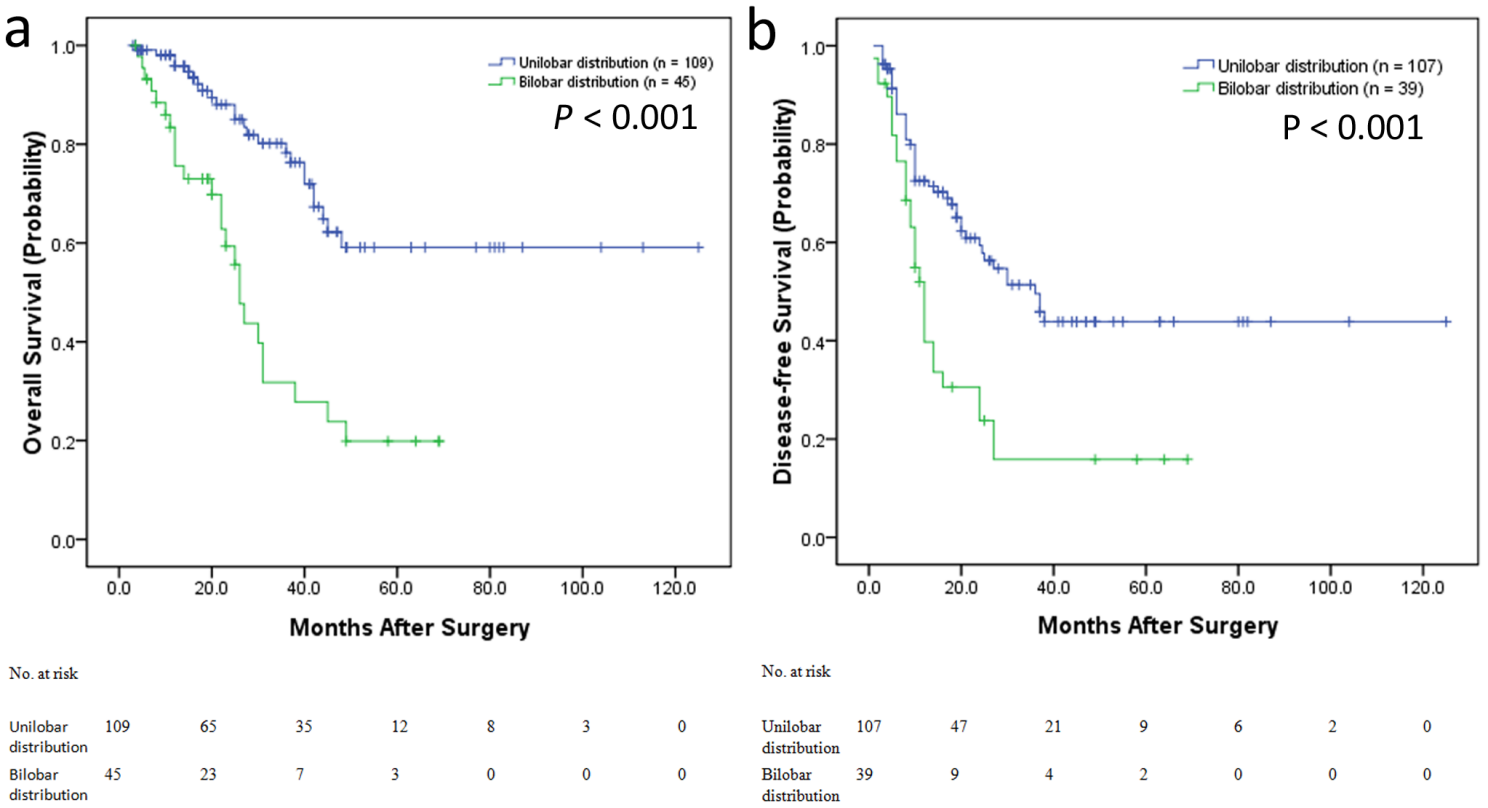
Kaplan-Meier estimates of the OS and DFS according to the tumor distribution (bilobar or unilobar). a) Kaplan–Meier curves illustrating the OS of bilobar distribution versus unilobar distribution patients. The OS of tumor bilobar distribution patients was significantly shorter than that of tumor unilobar distribution patients (P<0.001). b) Kaplan–Meier curves illustrating the DFS of bilobar distribution versus unilobar distribution patients. The DFS of tumor bilobar distribution patients was significantly shorter than that of tumor unilobar distribution patients (P<0.001).

As shown in Fig. 1, Kaplan-Meier survival analysis revealed that the OS (Fig. 1a) and DFS (Fig. 1b) of CRC patients with bilobar LMs were significantly poorer than those patients with unilobar LMs (P<0.001; P<0.001, respectively), indicating a crucial impact of LMs distribution on clinical outcome in patients.

### Operative Details and Perioperative Chemotherapy

A total of 7 patients (4.5%) underwent resection of extrahepatic metastases, and pathologically identified margin involvement was noted in 8 patients (5.2%). A total of 46 patients (29.9%) had 60 complications as follows: ascites (11), subphrenic fluid (9), pleural effusion (8), wound infection and fat liquefaction (6), small bowel obstruction (5), pneumonia and atelectasis (5), intra-abdominal infection (3), hemorrhage/hematoma (3), transient hepatic dysfunction (2), bile leakage (2), intestinal leakage (2) and others (4). All of the complications were successfully treated medically or by percutaneous drainage. Regarding adjuvant treatment, a total of 38 patients (24.7%) received preoperative chemotherapy; all of the patients received adjuvant chemotherapy. The routinely used chemotherapy regiments were FOLFOX, FOLFIRI and XELOX.

### Overall Survival Analysis

**Figure 2:**
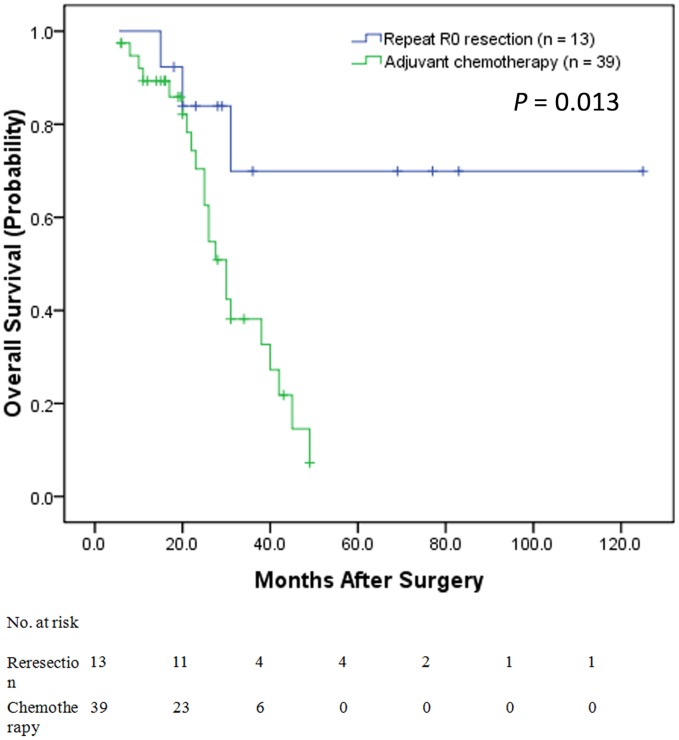
Kaplan–Meier curves illustrating the OS of patients with liver-only recurrence who underwent a repeat R0 resection versus those who underwent adjuvant chemotherapy only. Patients who underwent a repeat R0 resection had a good prognosis compared to those who underwent adjuvant chemotherapy only (P = 0.013).

Follow-up information was obtained for the 154 patients through July 2013. The median OS calculated from the time of diagnosis of the disease was 49 months. The 5-year OS rate was 46%. The median follow-up period was 36 months. At the last follow-up, 49 (31.8%) patients had died; 78 (50.6%) patients experienced tumor recurrence; 52 (35.6%) had recurrence in the liver only, 12 (8.2%) had recurrence in the lung only and 14 (9.6%) had recurrence in other sites. Among the 52 (35.6%) patients with recurrence in the liver only, 13 (25%) underwent a repeat R0 resection, and 39 (75%) received adjuvant chemotherapy. The former had a good prognosis, as determined with the Kaplan-Meier method (P = 0.013) (Fig. 2).

In univariate analyses, the following factors were significantly associated with poor OS: CEA level (>5 ng/ml), positive lymph node status, vascular invasion, nerve invasion and *BRAF* mutation in the primary tumor, number of metastases (≥4), bilobar distribution, and non-R0 resection of LMs. Multivariate analysis indicated that positive lymph node status, vascular invasion, *BRAF* mutation, bilobar LM distribution and non-R0 resection of LMs were significantly associated with poor prognosis. The details of the univariate and multivariate analyses are shown in Table 2.

**Table 2:** Univariate and multivariate analyses of associations between clinicopathological characteristics and overall survival after curative resection of liver metastases.

Prognostic factor	Univariate analysis			Multivariate analysis			
	HR	95% CI	P	HR	95% CI	P	β
Age (>60:≤60)	0.644	0.346–1.198	0.165				
Sex (Female:Male)	1.378	0.785–2.417	0.264				
Primary tumor site (Rectum:Colon)	1.288	0.720–2.307	0.394				
Histological type (Mucinous adenocarcinoma: Adenocarcinoma)	0.846	0.360–1.990	0.702				
Tumor differentiation (Well, moderate: Poor and others)	1.422	0.801–2.524	0.230				
Primary tumor (T) stage (T3,T4:T1,T2)	1.596	0.387–6.576	0.517				
Primary nodal (N) stage (N1,N2:N0)	3.336	1.617–6.885	0.001	4.108	1.907–8.853	<0.001	1.413
Tumor deposits (Positive:Negative)	1.688	0.962–2.962	0.068				
Vascular invasion (Positive:Negative)	2.358	1.121–4.555	0.011	2.928	1.410–6.080	0.004	1.074
Nerve invasion (Positive:Negative)	2.384	1.055–5.388	0.037				
CEA (>5 ng/ml:≤5 ng/ml)	2.109	1.050–4.238	0.036				
*KRAS* status (Mutated: Wild type)	1.494	0.835–2.672	0.176				
*BRAF* status (Mutated: Wild type)	3.232	1.498–6.976	0.003	2.531	1.102–5.811	0.029	0.928
No. of LMs (≥4:≤3)	3.988	2.094–7.595	<0.001				
Tumor distribution (Bilobar:Unilobar)	3.522	2.003–6.193	<0.001	2.331	1.265–4.294	0.007	0.846
Size of LM (≥5 cm:<5 cm)	1.489	0.808–2.744	0.202				
Resection margin (R1:R0)	12.381	4.969–30.847	<0.001	6.992	2.066–23.661	0.002	1.945
Extrahepatic metastases resection (Yes: No)	1.903	0.590–6.136	0.282				
Chemotherapy (Postoperative:Perioperative)	0.959	0.527–1.746	0.891				

**Figure 3:**
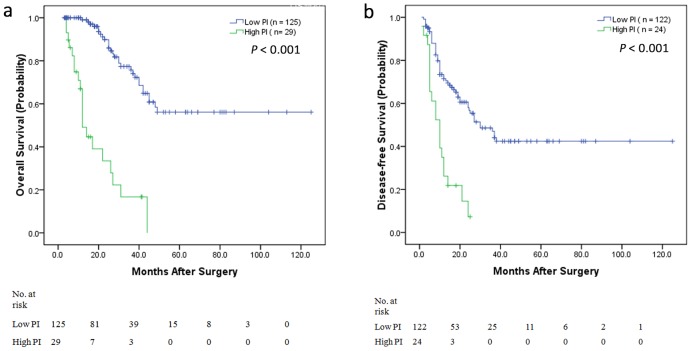
Kaplan-Meier estimates of OS and DFS from categorized analysis. a) OS categorized by high prognostic index (PI) and low PI. Patients with low PI had longer OS, whereas those with high PI had shorter OS (P<0.001). b) DFS categorized by high PI and low PI. Patients with low PI had longer DFS, whereas those with high PI had shorter DFS (P<0.001).

Based on the results of multivariate analyses, the PI for OS was calculated as follows: PI = (1.413×positive lymph nodes) + (1.074×vascular invasion) + (0.928×*BRAF* mutation) + (0.846×bilobar LM distribution) + (1.945×non-R0 LM resection) (β values was shown in Table 2). Kaplan-Meier estimates were calculated according to the PI value, and the cut-off point was the third quartile. A high PI (>5.978) was found to be highly predictive of the short-term outcome (P<0.001) (Fig. 3a). The 5-year OS rate for patients with low PI (≤5.978) was 55%, whereas the 5-year survival rate for patients with high PI (>5.978) was 0%.

### Disease-free Survival Analysis

The 5-year DFS of the 154 CRLM patients was 35%. In univariate analyses, tumor deposits, *BRAF* mutation, number of LMs (≥4), bilobar LM distribution and extrahepatic metastases resection were significantly associated with shorter DFS. Multivariate analysis indicated that tumor deposits, *BRAF* mutation and bilobar LM distribution were significantly associated with shorter DFS. The details of the univariate and multivariate analyses are shown in Table 3.

**Table 3:** Univariate and multivariate analyses of associations between clinicopathological characteristics and disease-free survival after curative resection of liver metastases.

Prognostic factor	Univariate analysis			Multivariate analysis			
	HR	95% CI	P	HR	95% CI	P	β
Age (>60:≤60)	0.671	0.408–1.104	0.116				
Sex (Female:Male)	1.529	0.971–2.409	0.067				
Primary tumor site (Rectum:Colon)	1.313	0.819–2.104	0.258				
Histological type (Mucinous adenocarcinoma: Adenocarcinoma)	0.620	0.298–1.292	0.202				
Tumor differentiation (Well, moderate: Poor and others)	1.423	0.898-2.255	0.133				
Primary tumor (T) stage (T3,T4:T1,T2)	1.539	0.484–4.893	0.465				
Primary nodal (N) stage (N1,N2:N0)	1.611	0.991–2.620	0.054				
Tumor deposits (Positive:Negative)	2.149	1.360–3.396	0.001	2.076	1.304–3.306	0.002	0.730
Vascular invasion (Positive:Negative)	1.482	0.828–2.652	0.485				
Nerve invasion (Positive:Negative)	1.453	0.722–2.926	0.295				
CEA (>5 ng/ml: ≤5 ng/ml)	1.429	0.868–2.352	0.160				
*KRAS* status (Mutated: Wild type)	1.509	0.941–2.419	0.088				
*BRAF* status (Mutated: Wild type)	4.400	2.263–8.553	<0.001	3.514	1.791–6.896	0.000	1.257
No. of LMs (≥4:≤3)	2.333	1.251–4.351	0.008				
Tumor distribution (Bilobar:Unilobar)	2.343	1.465–3.749	<0.001	2.325	1.443–3.746	0.001	0.844
Size of LM (≥5 cm:<5 cm)	1.420	0.872–2.312	0.159				
Extrahepatic metastases resection (Yes: No)	2.670	1.068–6.675	0.036				
Chemotherapy (Postoperative:Perioperative)	1.108	0.663–1.852	0.696				

The PI for DFS was calculated as follows: PI = (1.257×*BRAF* mutation) + (0.844×bilobar LM distribution) + (0.730×tumor deposits) (β values was shown in Table 3). Kaplan-Meier estimates were calculated according to the PI value; the cut-off point was the third quartile. A high PI (>2.945) was found to be highly predictive of the short-term outcome (P<0.001) (Fig. 3b). The 5-year DFS rate for patients with a low PI (≤2.945) was 41%, whereas the 3-year survival rate for patients with a high PI (>2.945) was 5%.

### Subgroup Analysis for Colon and Rectal Cancer

**Figure 4:**
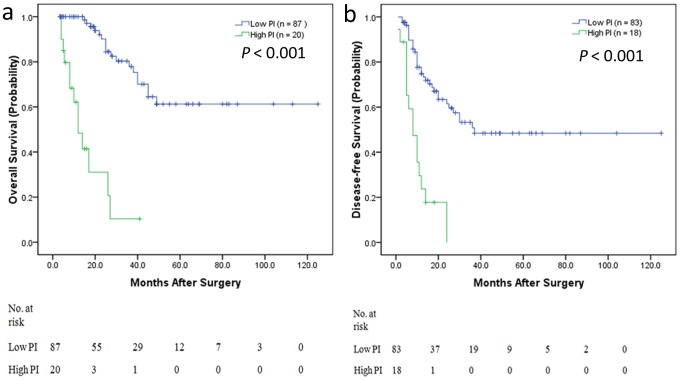
Kaplan-Meier estimates of OS and DFS from categorized analysis in colon cancer patients. a) OS categorized by high prognostic index (PI) and low PI. Patients with low PI had longer OS, whereas those with high PI had shorter OS (P<0.001). b) DFS categorized by high PI and low PI. Patients with low PI had longer DFS, whereas those with high PI had shorter DFS (P<0.001).

**Figure 5:**
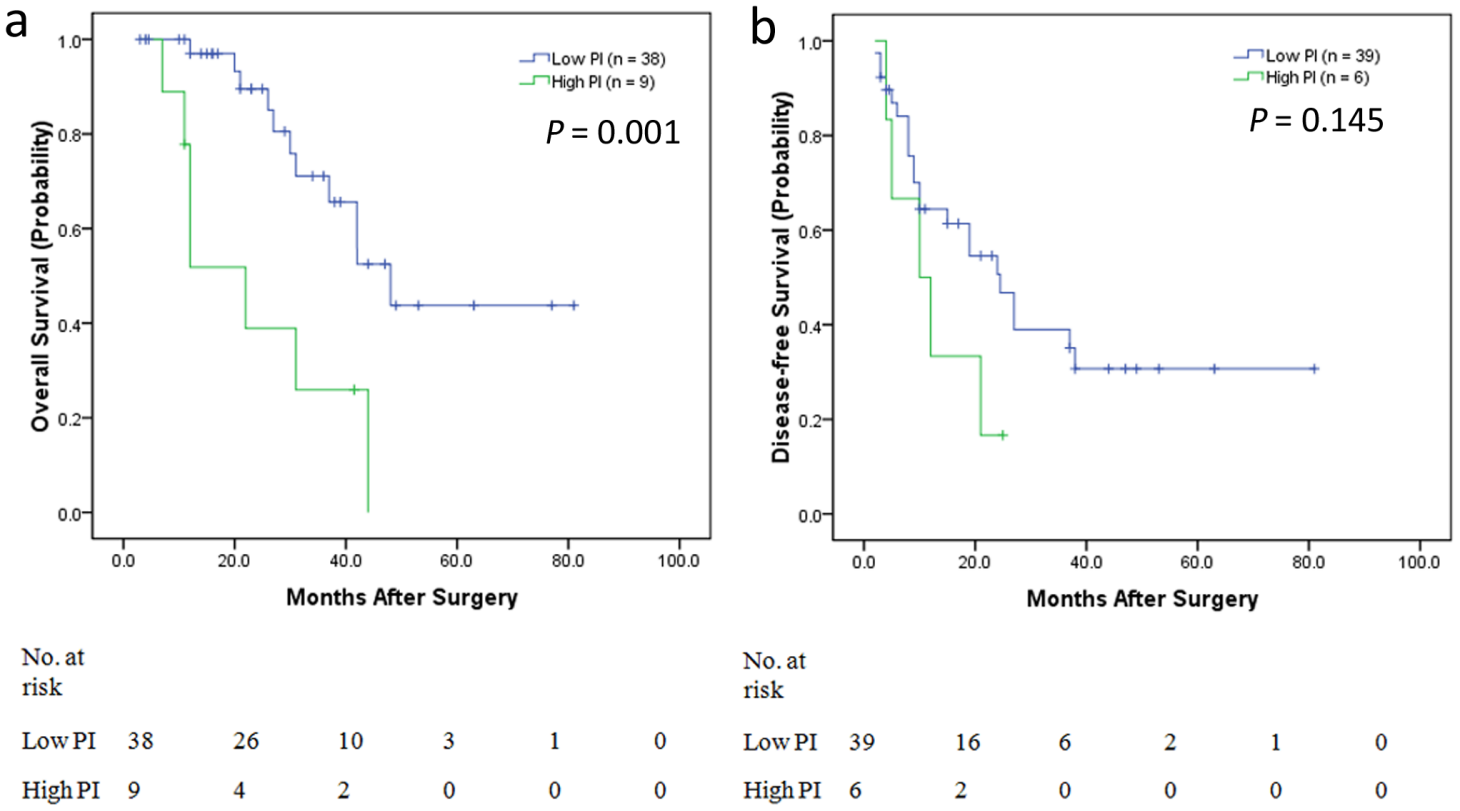
Kaplan-Meier estimates of OS and DFS from categorized analysis in rectal cancer patients. a) OS categorized by high prognostic index (PI) and low PI. Patients with low PI had longer OS, whereas those with high PI had shorter OS (P<0.001). b) DFS categorized by high PI and low PI. The OS of high PI patients was not significantly shorter than that of low PI patients (P = 0.145).

In consideration of the heterogeneity between colon and rectal cancer, we performed the categorized analysis according to high and low PI value for colon and rectal cancer patients respectively. Kaplan-Meier estimates were calculated according to high and low PI value. In colon cancer patients, a high PI (>5.978) was found to be highly predictive of the short-term OS (P<0.001) (Fig. 4a) and also high PI (>2.945) was found to be highly predictive of the short-term DFS (P<0.001) (Fig. 4b). In rectal cancer patients, a high PI (>5.978) was found to be highly predictive of the short-term OS (P = 0.001) (Fig. 5a), but high PI (>2.945) was not found to be highly predictive of the short-term DFS (P = 0.145) (Fig. 5b).

## Discussion

In recent decades, the strategy of delayed hepatectomy has gained popularity and has been established as the standard surgical practice. Some authors hold the view that simultaneous resections may increase the rate of postoperative complications [17]. Given that the morbidity and mortality associated with hepatectomy have decreased substantially over the past 20 years, the classic paradigm of a staged operation for SCRLMs has been questioned. Specifically, given the improved feasibility and safety of major hepatic resections, some investigators have suggested that the simultaneous approach to SCRLMs may be preferable. Other large studies, including meta-analyses [3], [4], [8], [9], have also shown that simultaneous resections are not associated with increased rates of hepatic or colon complications compared with delayed resection. Finally, as demonstrated by some large studies and meta-analyses examining the timing of hepatectomy for patients with SCRLMs, long-term OS and DFS are similar between the simultaneous and delayed resection groups [3], [4], [18]. In our study, the median overall survival was 49 months, the 5-year OS rate was 46%, and the 5-year DFS was 35%. These findings are in agreement with present studies [3], [4], [8], [9], [11], [19].

Recently, Abbott AM et al [18] found significant predictors of short outcomes for CRLMs about complications, mortality, and prolonged length of stay including age >70 years, male gender, nonprivate health insurance, and Elixhauser score. However, the most important problem is identifying determinants of the long-term outcome. Few studies have examined factors that are prognostic of OS and DFS following synchronous resection, and the sample sizes were very small [10], [19]–[22]. Most studies have used a Clinical Risk Score (including nodal status of the primary tumor, a disease-free interval from discovery of the primary tumor to discovery of LM<12 months, >1 tumor, a preoperative CEA level >200 ng/ml and size of the largest tumor >5 cm) [20] and the Basingstoke Predictive Index (including >3 hepatic metastases, node-positive primary disease, a poorly differentiated primary tumor, presence of extra-hepatic disease, tumor diameter ≥5 cm, CEA level >60 ng/ml and positive resection margins) for risk- categorizing patients who may benefit from intensive surveillance as well as for the selection of adjuvant therapy and trials [21]. However, there were more MCRLMs in these studies, and the prognostic factor “discovery of liver metastasis of <12 months” is unsuitable for predicting recurrence in SCRLM patients after simultaneous resection, as the OS and DFS of SCRLM patients are shorter than those of MCRLM patients. Furthermore, additional emphasis is now focused on tumor molecular stratification. Thus, the Clinical Risk Score must be updated. In our study, we found significant independent predictors of short OS, including positive lymph node status, vascular invasion, *BRAF* mutation, bilobar LM distribution and non-R0 resection of LMs. We determined the PI using these predictors, and a high PI (>5.978) was found to be highly predictive of short OS (P<0.001). We also performed categorized analysis using tumor deposits, *BRAF* mutation and bilobar LM distribution for DFS, and we found that a high PI (>2.945) was highly predictive of short-term DFS (P<0.001). We also performed the categorized analysis according to high and low PI value for colon and rectal cancer patients respectively. In colon cancer patients, a high PI was found to be highly predictive of the short-term OS and DFS. In rectal cancer patients, a high PI was found to be highly predictive of the short-term OS, but high PI was not found to be highly predictive of the short-term DFS, because of the small sample of rectal cancer.

In contrast to previous series, the number of LMs [10], [20]–[22] and the size of LMs [20], [21] were not independent predictors in our study. We examined different cut-offs for number and size in the univariate analyses, and we found that the hazard ratio of number of LMs (≤3; ≥4) and size of LMs (<5 cm; ≥5 cm) was maximal, in agreement with the above studies [10], [20]–[22]. Therefore, we preliminarily concluded that if R0 resection is achieved, the number and size of LMs do not conclusively influence OS; however, additional evidence is needed. In many studies, a high preoperative CEA has been associated with poor survival, particularly at higher cut-off levels [11], [19]–[21]. However, our study and other studies [10], [22] have failed to show that CEA is a significant prognostic factor, which could partially be due to the use of different cut-off values. In fact, we examined different cut-off values in univariate analyses and found that the hazard ratio of CEA >5 ng/ml/<5 ng/ml was maximal.

We first found that bilobar LM distribution and *BRAF* mutation of the primary tumor are independently associated with worse OS and DFS, respectively. Very few studies have explored the predictive role of bilobar distribution. In our study, OS and DFS of CRC patients with bilobar LMs were significantly poorer than those patients with unilobar LMs, indicating a crucial impact of LMs distribution on clinical outcome in patients.

*KRAS* and *BRAF*, which function downstream of the epidermal growth factor receptor (EGFR) signaling pathway, play important roles in the initiation and progression of CRC. Mutant *KRAS* is a predictor of resistance to EGFR monoclonal antibodies; thus, mutations in this gene are useful markers in clinical practice for patients with metastatic CRC [23]. Other studies have reported that *BRAF* mutations appear to be prognostic rather than predictive, as patients with metastatic CRC who do not receive cetuximab also have markedly reduced survival when their tumors harbor a *BRAF* mutation [24], [25]. Therefore, it is essential to include these two genes in studies attempting to identify prognostic predictors for SCRLM. In the current study, we determined that *BRAF* is an independent predictor of both OS and DFS.

Despite the theoretical advantages of neo-adjuvant chemotherapy, including the eradication of microscopic neoplastic foci, practical evidence for its benefit in patients with resectable SCRLM is weak. According to the results of the EORTC 40983 randomized trial, perioperative chemotherapy improves the outcome of resectable CRLM patients [26]; however, the control group in this trial only underwent surgery without adjuvant chemotherapy. Our study and some retrospective series specifically focused on resectable synchronous metastases have failed to demonstrate any survival advantage in patients receiving neoadjuvant treatments compared to adjuvant chemotherapy [27], [28]. Reddy SK et al [27] suggested that chemotherapy administered after but not before SCRLM resection is associated with improved DFS and OS. Another study including patients with SCRLMs and MCRLMs demonstrated that the effects of pre- and post-operative chemotherapy are indistinguishable [29] and that chemotherapy administered after but not before resection is associated with a good prognosis [30]. Prospective randomized trials are needed to determine the optimal timing of chemotherapy.

## Conclusions

Despite the limitations of the patient data showing a fair amount of early censoring, based on our preliminary data, we can make a conclusion that simultaneous resection of SCRLM may produce good long-term outcomes. Patients with low PI have longer OS and DFS, while those with high PI have shorter OS and DFS. Thus, patients with high PI may be provided more aggressive treatment and intensive surveillance, This model needs further validation.
